# Diagnostic and Therapeutic Potential of Selected microRNAs in Colorectal Cancer: A Literature Review

**DOI:** 10.3390/cancers17132135

**Published:** 2025-06-25

**Authors:** Grzegorz Sychowski, Hanna Romanowicz, Wojciech Ciesielski, Piotr Hogendorf, Adam Durczyński, Beata Smolarz

**Affiliations:** 1 Laboratory of Cancer Genetics, Department of Pathology, Polish Mother’s Memorial Hospital Research Institute, Rzgowska 281/289, 93-338 Lodz, Poland; 2 Department of General and Transplant Surgery, Medical University of Lodz, 90-419 Lodz, Poland; wojciech.ciesielski@umed.lodz.pl (W.C.);

**Keywords:** microRNA, colorectal cancer, cancer, biomarkers

## Abstract

Despite numerous studies, colon cancer is still one of the leading cancers in the world, both in terms of incidence and mortality. One of the most important methods of monitoring and studying changes in cancer is the use of biomarkers in the form of microRNA. Their remarkable stability and detectability in body fluids make them promising diagnostic and prognostic biomarkers. This review highlights selected deregulated miRNAs in colorectal cancer, including miR-21, miR-29b, miR-148a, miR-149, miR-155, miR-194, and miR-200b and lesser known ones such as miR-6803-5p or miR-4772-3p. Deregulation of their expression in colon cancer is associated with abnormal cell proliferation, migration, changes in the microenvironment or response to treatment, caused by their involvement in such pathways as Wnt/β-catenin, PI3K/Akt or EGFR. Additionally, we explore their therapeutic potential, particularly their ability to enhance tumour sensitivity to chemotherapeutic agents like 5-FU and oxaliplatin. The purpose of this review is to emphasize the growing relevance of miRNAs as both non-invasive diagnostic tools and potential therapeutic targets in colorectal cancer management.

## Simple Summary

Colorectal cancer remains one of the most common and deadly cancers worldwide. A promising area of research focuses on microRNAs—small regulatory molecules found in body fluids. Their stability and detectability make them valuable as non-invasive biomarkers. In this review, we describe several key microRNAs that are altered in colorectal cancer, including both well-known ones like miR-21 and less studied ones like miR-6803-5p. The influence of these particles on tumour growth, spread, and treatment response and their involvement in important cell signalling pathways is discussed. We also provide therapeutic and prognostic insights on the selected miRNAs based on current knowledge.

## 1. Biogenesis and Mechanisms of miRNA Action

MicroRNAs are a group of non-coding, single-stranded RNAs of about 20 nucleotides in length that are highly evolutionarily conserved between different groups of organisms [1]. 

In the 30 years of research conducted since the discovery of miRNA in 1993 in *Caenorhabditis elegans* [*C. elegans*] [2], it has been possible to demonstrate their broad impact on the regulation of gene expression. The basic mechanism of miRNA action is the inhibition of expression by binding to a given gene. This action is possible only after miRNA binds to one of the four argonaut proteins (Ago—all proteins of this type have four domains: N-terminal, PIWI, MID and PAZ), which results in the formation of the basic functional miRISC complex (miRNA-Induced Silencing Complex). It recognizes target mRNA thanks to the seed sequence, located two to seven nucleotides from the 5′ end. In addition, it interacts with PABP, which recognizes mRNA from the 3′ side and takes part in the formation of mRNA loops in the canonical translation process, and TNRC6/GW182, which as a scaffold protein mediates between Ago and PABP [3]. Thanks to them, the complex can interfere with the formation of the mRNA loop. MiRISC also disrupts the recognition of the 5′ cap by eIf4E and inhibits the recruitment of the 40S and 60S ribosomal subunits, which prevents initiation. Translation inhibition can also occur due to the induction of mRNA decay, in which process Ago and PABP lead to deadenylation of the mRNA, which becomes susceptible to decapping by the DCP1–DCP2 complex. After its loss, the mRNA can be recognized by exonucleases (Xrn 1 5′-3′) and its degradation can occur. The protein product itself can also be degraded on an ongoing basis, during its formation in the ribosome, if the miRNA complex has joined during elongation. miRISC is also able to induce degradation of the ribosome itself or detach it from the mRNA. The decision of whether an mRNA is degraded or only translation is interrupted depends on the level of complementarity of the miRNA binding to the target 3′ UTR sequence [4]. Studies on this complex report its high heterogeneity depending on the state and type of cell, which translates into a high number of functions of the complex [1,5]. miRNA itself is also characterized by variability due to the way it is created and the genes that encode it. MiRNAs are often encoded by polycistronic genes—more than half of microRNAs come from gene clusters dependent on a single promoter [6]. The transcription of microRNA genes is carried out by RNA polymerase II or polymerase III (POL II or POL III). Its product is a pri-miRNA 70 nucleotides long, with single-stranded ends, a double-stranded target miRNA segment, and a loop. The free ends are cut off in the nucleus by the microprocessor—the DGCR8 complex and DROSHA, which determines a double-stranded segment 11 base pairs long. Studies also report many alternative functions of this complex [7]. The transcript thus transformed by it, already called pre-miRNA, is exported from the nucleus by exportin 5 (Exp5) through nuclear pores. In the cytoplasm, further processing takes place in the DICER endonuclease complex (RNase III type) with the Hsp90 chaperone, which consists in disconnecting the apical loop and creating a double-stranded fragment consisting of the 3p and 5p strands of miRNA. Then, with the help of Hsp90 and HSC70, this duplex goes to Argonaut. The Ago protein separates the leading strand (which then becomes a mature miRNA) from the passenger strand, which is removed. Which one will be degraded depends on their thermodynamic stability and the preference of the MID domain of the Ago protein, which binds more strongly to uracil and cytosine than to other residues on nucleotides [8]. Ago with a single strand of miRNA is a mature silencing complex. The miRNA in it functions by recognizing the target mRNA based on the complementary 3′ UTR sequence and binding to it. The remaining passenger strand, however, is not always the 3p strand, although some miRNAs preferentially originate from the 5p band due to their structure. In addition, the removed passenger strand from one complex is able to reach the other and form an independent, functional complex [8]. For this reason, miRNA targets and functionality are often considered separately for the 5p and 3p transcripts, as these are complementary strands but, consequently, have different mRNA sequence, stability, and targets [9].

MiRNA can also be produced via non-canonical pathways, for example, MiR-451 bypassing DICER, where Argonaut 2 cleaves and degrades the passenger strand [10]. The RISC complex also receives fragments of tRNA that were created by DICER and angiogenin, through incorrect recognition of the tRNA loop as a substrate. Also, fragments of snoRNA (small nucleolar RNA) created as a result of incorrect processing by DICER are able to bind to Ago and form a functional RISC complex [11].

MiRNA is detected in the whole cell and in all tissues. It is also found in body fluids such as blood, plasma, saliva or even milk, as a result of leakage from damaged cells or apoptotic cells. They can also be secreted intentionally and actively using a protein complex or together with membrane vesicles, exosomes. Studies have proven that miRNAs are very stable molecules [12], and their durability in body fluids is supported by their complexation with proteins such as Ago2 or HDL—high-density lipoproteins [13]. The results of the studies also proved the resistance of miRNA to high temperatures, pH changes, and to embedding in paraffin and formalin. However, it was found that these methods have a negative impact on the stability of miRNA, which is especially visible in sequencing- based studies [14]. The data also report a negative impact of repeated freezing and thawing on the amount of miRNA in blood samples [15]. In healthy individuals, the expression level of many miRNAs usually remains constant over time [16]. High stability and ease of acquisition thanks to liquid biopsy techniques allow for their easy examination, and their expression pattern is a valuable picture of the patient’s health status.

The levels of detected miRNA in exosomes and freely circulating are different, which may result from the increased stability of acids enclosed in exosomes and depend on the type of miRNA or the patient’s condition [17,18].

## 2. Techniques and Challenges in miRNA Detection

Currently, there is no standardized diagnostic protocol based on miRNA, but most research studies use qPCR, Northern blot, chromogenic or fluorescent ISH, microarrays, and RNA sequencing techniques [13,16,19].

QRT-PCR is currently the most commonly used due to its relatively high throughput, low equipment costs, high sensitivity, and the multitude of reverse transcription methods, amplification (based on LAMP loops or RCA rolling circle), probes, and dyes developed over the years. However, there are still uncertainties regarding the veracity of the obtained results [20] and ways to design starters and normalize data [21]. Normalization to reference genes or to the absolute amount of RNA in the sample is suggested, as this method is more reliable but also more laborious [22]. Attempts have also been made to develop a bioinformatic method to normalize results to multiple patient parameters in studies of circulating plasma miRNA [23]. Another emerging issue is the difference between standardization of plasma, blood, and saliva samples. Systematic reviews highlight that the lack of uniform procedures remains one of the major barriers to the implementation of miRNA-based liquid biopsy in routine diagnostics and research protocols. The normalization factors such as endogenous genes or spike-in controls are especially needed for highly accurate diagnosis [24]. There are also ongoing works and considerations on the optimal selection of reference genes in miRNA studies [25,26]. The data indicate that methods that omit the reverse transcription step are characterized by higher assay robustness and therefore are more reliable [27]. These include methods such as SplintR-rPCR, which uses the viral SplintR ligase (NEB, Ipswich, MA, USA) and qPCR, or miREIA (Bio Vendor, Brno, Czech Republic)—miRNA analysis based on an immunological analysis similar to the ELISA analysis protocol [28].

Digital PCR is a refined version of qPCR utilizing oil droplets to perform multiple PCR reactions and fluorescence measurements at once. Due to this, it is more suited to the assessment of nucleic acids with low initial concentrations. This method can be used to assess the miRNA panel profile from stool as a colorectal cancer screening method [29] or as a diagnostic and prognostic tool, by assessing plasma or FFPE tissue-derived miRNAs [30,31]. The recently developed multiplex method can improve clinical applications of dPCR to a great extent thanks to fast and reliable assessment of six microRNAs simultaneously [32].

Northern blot uses agarose gels and nitrocellulose membranes and allows for the detection of miRNA fragments in the tested sample without specialist equipment. A wide range of probes and hybridization methods on membranes allows for its adjustment to the needs of the experiment. However, it is a laborious, low-throughput technique and does not detect low concentrations of miRNA [21].

In situ hybridization methods do not require RNA isolation, and therefore, it is possible to determine the location of miRNA in the cell. Chromogenic hybridization (CISH) allows for visualization of miRNA location using basic laboratory equipment such as a light microscope and probes labelled with biotin or digoxigenin [33]. It is also a cheaper and faster method than fluorescence hybridization (FISH), which requires a microscope with appropriate lighting and filters. In addition, FISH has a lower ability to detect a small number of miRNA copies [34]. Fluorescent dyes also tend to lose their properties over time. The use of LNA (locked nucleic acid) probes effectively and significantly increases the specificity of miRNA detection using ISH techniques by increasing the melting temperature of the tested sequence or probe, which positively affects the stability and efficiency of miRNA detection [35].

Next-generation sequencing (NGS) and microarrays are high-throughput methods. MiRNA arrays are based on a set of probes of different lengths anchored to a substrate, which, when hybridized with the tested labelled sequence, give a fluorescent signal received by the detector. However, they can give false results due to nonspecific binding of probes to transcripts. This is due to the variable melting temperature of different miRNA strands, due to different GC pair content [36], or sequences that are too similar to each other [21].

NGS systems show better performance than microarrays while maintaining similar accuracy [37]. Both of these methods are burdened with high costs, due to specialized equipment and the required high quality of the input miRNA. The single-cell sequencing methods significantly improved the understanding of TME, thanks to their unrivalled resolution. This method allowed to distinguish molecular subtypes of colorectal cancer and to create a new classification system—Consensus Molecular Subtypes (CMS). The scRNA-seq can create a tremendous amount of data, and analyzing them can be a challenging task [38]. To address this problem, new algorithms, metrics, and tools are developed to improve the analyses, for example, a diagnostic panel of fecal extracellular vesicle microRNA signatures with AI support [39] or a metric based on LASSO penalized Cox model improving survival predictions [40].

Even more advanced technology, such as single-molecule sequencing, allows researchers to track a single molecule of miRNA in the cell. Thanks to this method, Kobayashi and Singer described RISC complex functions in situ in great detail, and showed that it binds to mRNA immediately after it leaves the nucleus; then after 30 min, it inhibits its transcription, and finally, after 60 min, it induces its degradation [41].

## 3. Diagnostic Utility of miRNA in Colorectal Cancer

Colorectal cancer, despite intensive research on its treatment and diagnostics, is still the third most frequently diagnosed cancer in the world, and the number of deaths caused by it places it in second place among other cancers [34]. Studies indicate a significant environmental influence on the likelihood of developing this cancer, especially alcohol consumption, smoking, a sedentary lifestyle, and a poorly diversified diet rich in fats [35]. Such a lifestyle is becoming more common with the ongoing economic development, which translates into higher and higher disease statistics among younger (<30–40 years old) people. Retrospective analyses show that generations born after 1960 have an increasingly higher chance of developing CRC [42].

Bacterial infections are also a significant environmental factor in likelihood of developing CRC. The research showed that there is a dialogue between microbiome, intestine, and cancer cells. The mice infected with *L. monocytogenes* had downregulated mir-200b, miR200c, miR148a, miR-194 while in mice without the microbiome removed the miR-378 was elevated and miR-194 was decreased [43]. *F. nucleatum* proved to be directly responsible for metastasis formation and suppression of T and NK cell response [44].

The common method of treating colon cancer is surgical resection of the local tumour, in later stages of the disease supplemented with pre- or postoperative chemotherapy [45]. Due to the relatively long period of transformation of harmless polyps into cancer, the most effective results are brought by prevention and regular, widely available screening tests—colonoscopy and occult blood tests [46,47].

### Therapeutic Potential of miRNA in Colorectal Cancer

The miRNA therapies generally relay on synthetic mRNA mimics or miRNA inhibitors (antisense oligonucleotides blocking the activity of a target miRNA), which are delivered by an adequate vessel to the selected region to suppress the growth of tumour or metastasis, by upregulating tumour suppressor miRNAs like miR-194 or downregulating oncomirs such as miR-21 or miR-155 [48,49]. Despite numerous findings of potential therapeutic miRNA targets, there are few therapies utilizing those discoveries. Most of the current research is still in its preclinical trials or without administrative approval [50]. The most prominent issue preventing progress seems to be the adverse effects on healthy cells caused by unprecise miRNA delivery (off-target effect), causing immune response or mutations further contributing to tumour growth [51]. Another problem is the stability and bioavailability of miRNA in vivo, which must be resolved by a sufficient and precise method of delivering the miRNA mimics to selected cells [52]. Currently, the tested methods of delivering the miRNA mimics are polymeric nanoparticles, viral vectors, and lipid vesicles. The example of a polymeric nanoparticle vessel used in CRC treatment is the galactose-targeted calcium lipid phosphate nanoparticles used with miR-122 to prevent colorectal cancer liver metastases [53]. The lipid vessels on the other hand are simple and cheap to manufacture, non-toxic, and offer a variety of types: various lipid nanoparticles (composed of solid and liquid lipids) and liposome subtypes such as cationic liposomes, neutral liposomes, and ionizable liposomes. This high modification potential, specificity, and indifference to immune system makes both liposomes and nanoparticles some of the most promising and broadly used tools for miRNA therapy. Exosomes are also lipid nanoparticles, but contrary to others, they are derived from cells, and thus they offer perfect bioavailability and trigger no immune response. On the downside, the effective production of them is laborious, and the miRNA loading process is burdened with problems with miRNA-packaging enzyme–exosome compatibility [54]. In terms of viral vectors, there are three subtypes of viral vectors which can be distinguished: the adenovirus vectors, lentivirus vectors, and adeno-associated virus vectors. Each of them has its own weaknesses and advantages, which are mostly stability and transduction issues, which could possibly result in mutations and immunologic response [55]. All of the aforementioned methods are used to increase the stability of miRNA, the drug uptake from cells and the specificity, thus reducing potential toxic side effects [52]. Moreover, the modifications of miRNA itself are used, for example, 2′-O-methylation or incorporating LNA or additional nucleic acid structures to microRNA. Regardless of the drug delivery method used, the development of miRNA therapy is costly (hundreds of millions of dollars) and laborious, which delays the appearance of an effective, available, and affordable miRNA cancer therapy on the market [48].

## 4. Methods

The role of many miRNAs in colorectal cancer has been the subject of independent studies. This review aims to provide an overview of the current knowledge on selected CRC-related miRNAs studied in recent decades. The inclusion criteria were based on their functional involvement in key molecular processes and their potential as prognostic markers. To offer a comprehensive overview that encompasses both well-established and emerging microRNAs relevant to colorectal cancer, we included well-established miRNA such as miR-21 or miR-155, but also the ones which were described only in a few papers, making them potential novel research targets. The main characteristics of discussed miRNA are summarized in the Table 1. Their sequences are provided in the Supplementary Materials to highlight the variability and diversity within their families. The PubMed, Scopus, MDPI, and Google Scholar databases were searched for papers fulfilling those criteria, with keywords such as microRNA, miRNA, colorectal cancer, and biomarker.

Mir-21-5p—This was one of the first onco-miRNAs studied, and therefore the subject of many experiments and publications. Its gene is located on chromosome 17, from which both strands—5p and 3p—are derived. Its expression is deregulated in many diseases, and therefore, its specificity as a biomarker specific for colon cancer and other cancers is low. However, it is critical for many signalling pathways [94], and as such it is an important prognostic marker and indicator of disease progression [95]. However, it shows higher accuracy and specificity as a part of the miRNA marker panel [96]. It has been shown that in colorectal adenocarcinoma (COAD), its expression strongly correlated with the invasion, migration, and proliferation of COAD cells [97]. Through the TGF-β1 pathway, it is involved in the induction of pyroptosis in HCT116 colon cancer cell lines, in which its high expression is observed [56]. In turn, by affecting TGFβR2, miR-21 is able to modulate the cell adhesion system, as well as by promoting the formation of E-cadherin and catenins. There is also a correlation between CRC drug resistance and the expression of this miRNA [57]. 

MiR-21-3p—Studies show an overall lower presence of this transcript in cancer than in healthy tissue [98], but according to other studies, the difference in expression between peritumoral tissue and the tumour was not significant [56]. However, high levels of miR-21-3p were associated with better overall survival of patients. Expression of this miRNA decreases with T stage progression [98]. On the other hand, research conducted on the CRC cell lines (HCT16, HT29, Colo320 i SW480) showed that mir-21-3p can have oncogenic properties, and inhibiting its expression results in tumour growth and invasion suppression [99]. Those findings were also confirmed by research on natural mir-21-3p inhibitor—lnc RNA FAM30A—whose expression resulted in better survivability of CRC [100]. Interestingly, according to studies using the non-small cell lung cancer cell line PC9, miR-21-5p inhibitors negatively affected the expression of the 3p strand, but strict 3p strand inhibitors had no effect on the regulation of 5p strand expression [101]. The 3p strand sequence is less frequently detected in cells than the 5p strand.

**Figure 1:**
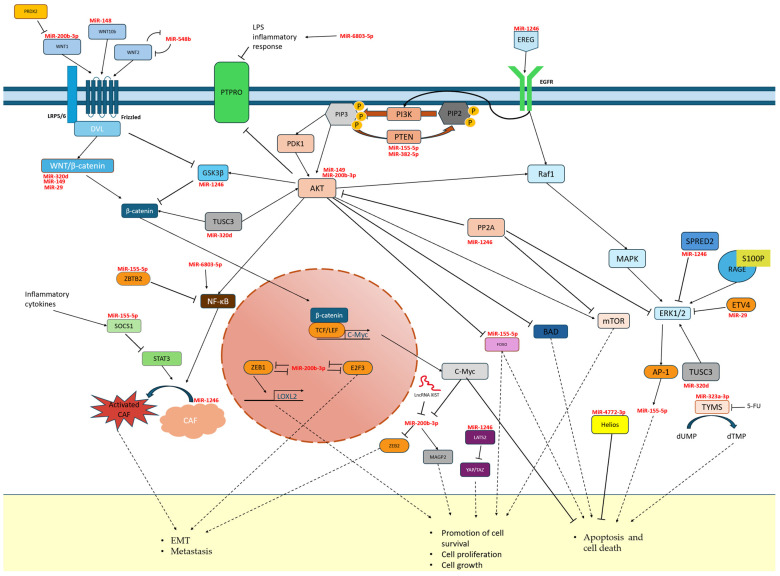
Pathways involved in CRC progression and microRNA inhibition targets involved in them.

MiR-29b—This microRNA is produced from the 3p strand of two genes, miR-29b-1 and miR-29b-2, located on chromosomes 7 and 1, respectively. Near these genes are located miR29a and miR29c, with which miR-29b1 and 2 share a promoter. The sequences produced from their 3p strand are practically identical to those of miR-29b [102]. In turn, 5p transcripts are much less common than the 3p sequence and differ significantly in structure. The miR-29b-3p sequence is responsible for, among other things, regulating the cell cycle and apoptosis. Studies have shown the role of miR29b as a repressor of colon cancer, by limiting Wnt, TGFβ (transforming growth factor β) or MAPK signalling, leading to reduced proliferation of tumour cells [58]. By suppressing the ETV4 transcription factor and thus the ERK pathway, miR29b is reducing tumour angiogenesis and EMT [103] (Figure 1). This makes it a potential therapeutic target in the treatment process [104,105]. It shows reduced expression in tissue and plasma in patients with CRC, and it is inversely proportional to the size of the tumour and stage of the disease [106]. However, age and gender do not affect the level of its expression, and postoperatively, it also increases to the same extent in all patients [107]; hence, its higher value was associated with better treatment prognosis. In a 2013 study, increased expression of miR-29b was found in colon cancer cells after exposure to American ginseng extract, which may indicate ginseng’s health properties [108]. Although Mir-29 levels are usually reduced in cancers, studies have shown an overregulation of miR-29a in metastatic CRC. The optimal sensitivity and specificity of miR-29b as a marker was achieved in combination with other methods and markers of colorectal cancer [59].

MiR-148 belongs to the miRNA 148/152 family. The UCAGUGCACUACAGAACUUUGU sequence is obtained from two genes—miR-148a located on chromosome 7 and miR-148b located on chromosome 12. In both cases, it arises from the 3p arm and occurs many times more frequently than the transcript from the 5p arm [109]. MiR-148a-3p is characterized by reduced expression in colorectal cancer [60] and colon adenocarcinoma cells (cell lines SW480 and SW620) [110]. The miR-148a-3p copy number was also negatively correlated with TNM tumour stage and the number of metastases [60]. Functionally, it has been evaluated as a tumour suppressor by inducing ferroptosis by limiting SLC7A11 expression. Additionally, it affects lipid peroxidation by inhibiting GPX4 expression. Higher levels of this microRNA inhibit CRC progression by initiating caspase 3-dependent programmed cell death [110]. The results of the 2019 study by Zheng et al. also confirm the involvement of miR-148a-3p in tumour escape from the immune system. Targeting the 3′UTR of the *CANX* gene (calnexin), it disrupts the process of synthesis of MHC I proteins—histocompatibility proteins presented on the cell surface for T cells. Their improper functioning results in the lack of recognition of cancer cells by the immune system and the lack of induction of the CD8 + response by T cells [61]. One of the studied targets of MiR-148a-3p is WNT10b, a member of the WNT gene family. Together with β-catenin, they are responsible for signalling pathways regulating the developmental processes of the organism and single cells, as well as stem cell transformations and pluripotency. It has been proven that high WNT10b expression, unrestricted by miR-148a-3p, is associated with a worse prognosis and may lead to tumour growth [60,111].

MiR-149-3p—Studies show that it is characterized by reduced expression in colon cancer. It is also much less common in the transcriptome than its 5p strand and, as a “passenger” strand, is functionally different, as in most miRNAs [112]. It is a potential tumour suppressor of CRC due to the inhibition of cancer cell motility by limiting the expression of CYBRD1. This protein, being an iron transporter to the cell, contributes to increased proliferation and reduced adhesion [62]. Studies have shown that inhibition of Wnt and AKT1 pathways by miR-149-3p and 5p increases drug susceptibility in cancers [112]. The increase in miR-149-3p level resulted in the induction of apoptosis in HCT116 cells, and its decrease limited the susceptibility to 5-FU. A negative correlation of lncRNA MAFG-AS1 with miR-149-3p expression was also detected, which indirectly, through the activation of HOXB8 (homeobox B8), influenced the increased proliferation of colon cancer cells (Figure 1) [63]. In 2021, Lin et al. proposed the use of miR-149-3p as a marker (sensitivity 76.74%, specificity 84.5%) of patients’ nutritional status for preoperative assessment of their health condition [113]. This miRNA, as a part of a five-microRNA panel, was also a reliable CRC biomarker derived noninvasively from stool [64].

MiR-155 is located on chromosome 21 in the region of the non-coding B cell integration cluster (BIC), currently called the MIR155HG gene. The most frequently present in the cell is miR155-5p, whose role and functions are well known. The 3p strand is expressed at a very low level, and due to difficulties in its detection, its biological functions are still unknown [65]. MiRNA-155 is involved in many regulatory and developmental processes, especially in erythrocytes and lymphocytes. It is considered one of the key regulators of the immune system. It also inhibits apoptosis and promotes proliferation in cancer cells via the PTEN/PI3K pathway (Figure 1), even when transferred exosomally. In addition, in cancers it regulates migration, invasion, and angiogenesis and contributes to the formation of cancer stem cells [114,115]. It is characterized by high prevalence [40] and high expression in cancers (including colorectal cancer), which is correlated with metastases, tumour progression, and poorer survivability. It is considered a typical oncomiR and a potential useful marker of cancer burden [67,114,115]. A feedback loop has been demonstrated with the key regulator of many processes—NF-κB—by inhibiting the activation of the Akt pathway. A nanoparticle-based method for CRC treatment using anti-miR-155 has also been developed [115]. The mir-155 is also strongly associated with inflammatory processes and responses to them, as it has been shown to be sensitive to some cytokines, and the Toll receptor and has been proven to play a key role in the functioning of B, T, and dendritic immune cells. In these cells, it is a functional antagonist of miR-146a [116]. Moreover, it has been shown that potential CRC therapy may also be based on elements of the S100P/RAGE signalling pathway, as it, together with miR-155, modulated and induced a neoplastic phenotype in healthy intestinal tissues [117]. The expression of this microRNA also increases in cancer cells following exposure to intense radiation. According to a 2017 study, this may result in the acquisition of radio resistance by cancer cells via disruption of the FOXO3a pathway, which is the target of miR-155 (Figure 1) [66]. This molecule may also contribute to the development of metastases by activating the chemokine receptor pathway CXCL12/CXCR7, whose connection with metastases has been proven by studies. This pathway affects miR-155, leading to phenotypic changes and activation of CAFs (cancer activated fibroblasts), which promote epithelial–mesenchymal transition and, consequently, metastasis [118].

MiR-194 belongs to cluster genes, which means that its promoter also affects the expression of another miRNA gene located nearby. In addition, the sequence from which mature transcripts are created occurs in humans in two copies—miR-194-1 on chromosome 1 in a cluster with miR-215 and miR-19-2 on chromosome 11 in a cluster with miR-192 [119]. Literature data report that both strands 3p and 5p have similar tumour-suppressive properties but are characterized by different regulatory pathways. The effects of overregulation of this miRNA include attenuation of tumour cell growth, migration, EMT, drug resistance, and proliferation. MiR-194-2-5p is a suppressor microRNA with reduced expression in colorectal cancer [68,120]. The strand is functionally related to the MAP4K4/c-JUN/MDM2 pathway, where it targets MAP4K4 and inhibits its expression. This results in inhibition of tumour cell proliferation, which is why it is a potentially important therapeutic target [120]. Studies also report its involvement in the induction and regulation of inflammation, depressive changes, and the development of addictions [121]. Its repressor is the regulatory protein HMGA2 (a non-histone chromosomal protein called high mobility group AT hook 2) [122]. One of the effector proteins is VAPA, which is responsible for regulating intracellular transport and maintaining the ER structure; regulation of this protein by miR-194 disrupts tumour growth [122,123]. 

MiR-194-3p—An important target in the tumour suppression process by miR-194-3p is KLK10 (kallikrein 10) [124], a serine protease with variable expression in cancer, whose role and mechanism of action are not yet fully understood, but according to some studies, it may act as a tumour suppressor in prostate cancer [116]. It has been shown that miR-194-3p is able to disrupt the functioning of colon cancer cells through interaction with KLK10 [124]. This transcript could also limit invasion, migration, and proliferation of CRC cells by inhibiting oncogenic TGFα. TP73-AS1 (lncRNA P73 antisense RNA 1T), in turn, acted inversely to miR-194-3p, simultaneously limiting the expression of miR-194-3p itself [70].

MiR-200b-3p is a member of the miR200 family, with suppressor genes located in two clusters on chromosomes 1 and 12. This group is involved in the regulation of key processes in many cancers and affects cell proliferation, invasiveness, and differentiation [71]. Tissue and cell line studies have shown reduced expression of miR-200b-3p in CRC relative to healthy tissues, and the level decreased with disease progression negatively affecting OS [125]. This may be due to its suppressive role in cancer cells by regulating the Wnt pathway by inhibiting Wnt1, which is associated with the formation and degradation of β-catenin, which is one of the signal transducers in the Wnt pathway in the cell (Figure 1) [126]. MiR-200b-3p is correlated with increased peroxiredoxin 2 (PRDX2) in CRC, which is responsible for reducing hydrogen peroxide and other reactive hydroxy peroxides. This enzyme, by inhibiting the activity of miR-200b-3p, promotes the development of the malignant phenotype of CRC cells. It also participates in the Wnt/β-catenin pathway, which affects c-Myc levels. A mechanism for the regulation of proliferation and invasiveness of colon cancer cells by the c-Myc/GSK3β/Akt2 pathway was also discovered. C-Myc bound the miR-200b-3p promoter, leading to ZEB2-dependant E-cadherin depletion and EMT initiation [127]. An inhibitory effect of miR-200b-3p on Akt2 was found, which through phosphorylation of GSK3β limited c-Myc, affecting its stability through phosphorylation of S62 and T58. Through these two regulatory pathways, miR-200b-3p is able to limit the proliferation and invasiveness of colon cancer cells and influence the resistance to chemotherapy [125]. The chemoresistance of CRC was influenced by βIII-tubulin (TUBBIII), whose inhibition by miR-200b-3p increased the susceptibility of cancer cells to oxaliplatin treatment [74]. In addition to the above-mentioned pathways involved in tumour growth, the pro-proliferative activity of microfibril-associated glycoprotein 2 (MAGP2), upregulated in tumours, has also been reported to be inhibited by direct binding of miR-200b-3p to its 3′ UTR region [73]. The suppressive effect of miR-200b-3p on tumour cell growth is also evident in the results of the 2023 study by Gong et al. It was noted that miR-200b-3p levels were significantly reduced in hypoxic cancer-associated fibroblast cells, as well as in exosomes derived from them. Both CAFs and their exosomes were able to promote changes in other CRC cells towards their increased malignancy and resistance to 5-FU treatment. Reversal of this effect was possible by reducing ZEB1 (a zinc finger protein involved in the EMT process [128]) and E2F3 (transcription factor 3 E2F, which plays a role in the cell cycle and is a key regulator of CSCs [129]), thanks to transfection of cells with agomir—artificial miR-200b-3p [130]. In colorectal cancer, the regulation of miR-200b-3p may also occur via long non-coding RNAs (lncRNAs), e.g., lncRNA XIST, which has a negative effect on the expression of miR200b-3p by directly binding to it [72].

MiR-204-5p—This is localized in the intron region of TRPM3 (cation channel protein) and is considered a tumour suppressor significantly downregulated in colorectal cancer. The main targets are RABB22 and CREB1, which mediates the inhibition of tumour growth, metastasis, and chemoresistance. It can be regulated by TRPM3 gene promoter methylation and by lnc RNA [131]. Another important pathway regulated by mir-204-5p is the glycosylation process, particularly the B4GALNT2 enzyme [76]. It is a glycosyltransferase downregulated in CRC, which results in worse overall survival, caused by the increased tumour growth and its invasiveness and formation of cancer stem cells [132]. The miR-204-5p and FOXD1 inhibits its expression, playing the role of oncomiR in this pathway [76]. The expression of miR-204-5p in colorectal cancer and its impact on survivability was studied, and the researchers showed a correlation between lowered miR-204-5p and poor survival of digestive system cancers [75]. Recently, a novel method of delivering the miR-204 to cancer cells in anti CRC therapy was developed. By modifying HEK293T cells, cell-derived miR-204-5p enriched exosomes with 7D12 nanobody, specific to EGFR on the surface of the EGFR+ cancer cells. This method offered high efficiency and specificity in delivering the transcript, resulting in promising therapeutic effects [77].

MiR-320—The miR 320 family undergoes a non-canonical Drosha-independent processing pathway, which is characteristic of miRNAs whose genes originate from mirtron regions or independent transcription units [7]. The maturation process of miR320, like all other microRNAs, requires Dicer, but it is independent of DGCR8, which recognizes pre-miRNA loops/hairpins and, together with Drosha, is an element of the nuclear microprocessor [133]. MiR-320a-3p is transcribed from a stem-loop precursor hsa-mir-320 gene, located on chromosome 8. Mature miR-320a-3p is more common than strand 5p, which according to miRbase can only arise from the 320a precursor [134]. Studies report reduced expression of this microRNA family in cancers [135], including colon cancer, but also gliomas and osteosarcoma, which was correlated with worse OS. Interestingly, in pancreatic cancer, it was characterized by increased expression [136]. The data also confirm an increase in expression levels in higher stages of CRC, which may be related to an attempt to defend cells transforming into cancer cells and acquiring a malignant phenotype [137]. The study results also showed a significant correlation between miR-320a levels and the number of CRC metastases [138]. According to Fang et al., the diagnostic accuracy of miR-320a measured in plasma in detecting early CRC is more than 90% [139]. Functionally, the miR-320 family has been designated as tumour suppressors because when upregulated, they inhibit cell proliferation and regulate chemoresistance through interaction with CDK6, Rac1 [140], FOX1M pathway, Wnt/β-catenin [141], SOX4, and FOXQ1. In CRC signalling, the possible targets are also HMGB3, MKI67, and ZWILCH, which are confirmed targets of the miR-320 family in cancers other than CRC [142]. MiR-320b, in turn, showed increased expression in patients with CRC and liver metastases. A positive effect on the levels of the above-mentioned proteins Rac1 and β-catenin was also reported [78]. Due to its stability, miR-320d is the suggested normalizer between cohorts and technical replicates in RT-qPCR studies [26]. Yufeng et al.’s studies on the role of miR-320d in EGFR-positive colon cancer cells showed that it is associated with TUSC3, which is upregulated in CRC. By inhibiting it, miR-320d contributes to limiting the proliferation, invasiveness, and EMT of the colon cancer (Figure 1) [79]. It also indirectly affects few pathways, because TUSC3 is involved in the Wnt pathway by interacting with β-catenin and MAPK and PI3k/Akt pathways due to Akt and ERK1/2 phosphorylation regulation [143,144].

MiR-323a-3p—The genes for miR-323a and b are located in one cluster of several other miRNAs on chromosome 14. Lee et al. propose to use this miRNA as a member of the miR-382-5p and miR-376a-3p miRNA panel, which shows high prognostic accuracy for lymph node metastasis in colorectal cancer [145]. Studies on this miRNA have shown that it has tumour-suppressive properties. Its expression levels are reduced in CRC cells. It inhibits the PI3K/AKT/GEK3B-Erk1/2 pathway by inhibiting EGFR/Erb3, which are membrane receptors responsible for signal transduction. This process is reducing the tumour’s acquired drug resistance to EGFR tyrosine kinase inhibitors (TKIs), such as gefitinib [80]. Signalling through EGFR occurs after its ligand-induced activation, which causes a conformational change and dimerization. Signalling pathways involving EGFR, such as the aforementioned PI3K/Akt or Ras/MAPK pathway, are responsible for regulating cell proliferation and survival. EGFR also plays a role in signalling cytokine-induced inflammation via the PI3K/Akt/NF-kB pathway [146]. Blocking EGFR/ErbB3 phosphorylation in the Pi3K/Akt pathway increases apoptosis and susceptibility to TKI treatment [80]. MiR-323a-3p can regulate the proliferation of colon cancer cells also by silencing NEK6, a protein responsible for the formation of the mitotic spindle. This leads to the cessation of cell growth, division, and the induction of apoptosis [81]. Another target for MiR-323a-3p is thymidylate synthase (TYMS). It is characterized by increased expression levels in many cancers including CRC and is responsible for the conversion of dUMP to dTMP and is the only provider of thymidylate for DNA synthesis. It is a key target for chemotherapeutic drugs such as 5-FU. Their metabolic product is FdUTP, which binds with TYMS, inhibiting its activity (Figure 1). No repair mechanism is available for repairing this error, thanks to which the drug has a cytotoxic effect [147]. Studies have shown that inhibition of TYMS via MiR-323a-3p increases the susceptibility of CRC cells to 5-FU treatment [82].

MiR-376—This is a microRNA whose family is located on chromosome 14 and whose sequences are placed sequentially on one strand of DNA. The results of the studies allow us to characterize them as miRNA with different activity. Mir-376a b and c found in plasma were characterized by reduced expression in gliomas and were associated with negative clinical features [148]. Studies conducted on tumour samples from CRC patients have shown reduced expression of miR-376b-3p compared to healthy intestinal epithelial tissue. SMAD proteins, key enzymes in the TGF-β pathway that can lead to metastasis, can be a target for miR-376b-3p. A significant correlation of miR-376b-3p levels with TNM tumour stage, number of metastases, and miR-552-5p expression has also been confirmed [84], which is increased in CRC and is believed to be associated with a worse prognosis for patients [83]. MiR-376b-3p and miR-654-5p showed high specificity and accuracy as markers useful for diagnosing and prognosticating the course of colorectal cancer, due to their significant correlation with the clinical features of the patient [84]. MiR-376 inhibits PRKD1, which by binding β-catenin may inhibit tumour growth [149]. LncRNA MEG3, in turn, shows onco-suppressive activity because it is able to block miR-376 by acting as a miRNA sponge [84,150]. Reduced expression of miR-376c in neoplastic cells was also observed in neuroblastoma cell lines. MiR-376c, by inhibiting the CCND1 gene, could limit their growth [151]. It also showed lower expression and onco-suppressive activity in oral squamous cell carcinoma and gastric cancer cells [152,153].

MiR-382-5p is located on chromosome 14, in the vicinity of miR-134 and miR-668, in the MEG9 gene, an anti-apoptotic lncRNA associated with genotoxic stress [154]. According to miRbase, the 5p strand is a more abundant transcript in cells than the 3p strand. Studies have reported strongly reduced expression of miR-382 in CRC tissues and cells and some other cancers, in which it was correlated with worse OS. Yao et al. showed that miR-382 targets KLF12, a transcription factor involved in the development of organisms, and HIPK3, which is involved in the regulation of metabolism and chemoresistance [85]. The results of the work prove the significant influence of cyclic RNAs on microRNA regulation, and therefore their important role in new formation and metabolism. CircRNA works on the principle of a microRNA sponge—in its structure it can have dozens of binding sites for various RNA sequences and proteins and transcription factors. These are also molecules resistant to RNAses, due to the lack of a 5′ cap and a poly-A tail on side 3, thanks to which they are present in the cell more than twice as long as their linear counterparts [155]. Cyclic RNA circ_0000467 via binding mir382-5p led to the malignant phenotype of frequently dividing and metastatic colorectal cancer cells [156]. Another oncogenic circRNA binding miR-382-5p is circ_0022340, which promotes tumour cell proliferation via the mir382-5p/ELK1 pathway [157]. Studies have shown the onco-suppressive effect of miR-382-5p on CRC cells by directly regulating erythrocyte nuclear factor 2-related factor 2 (NRF2) [87] and programmed cell death ligand 1 (PD-L1) [86]. In addition, miR-382-5p targets EN2, a homeobox protein engrailed-2, with oncogenic properties and characterized by increased expression in tumours [156]. MiR-382-5p can also inhibit tumour suppressor proteins, e.g., PTEN, which limits the activity of the PI3K/Akt pathway and leads to a reduction in the proliferation and migration of its cells (Figure 1). Circ_0008285 has the ability to sponge miR-382-5p and thus allows the activity of oncogenes and contributes to tumour growth [158].

**Figure 2:**
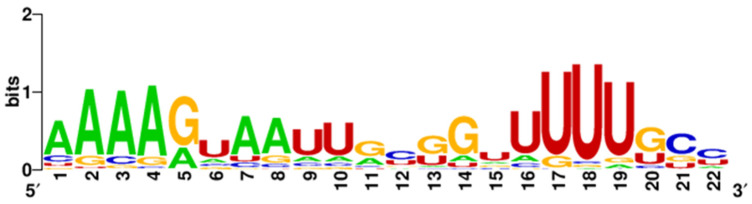
Variability in the sequence of the 5p strand in the miR-548 family. Created by WebLogo on: weblogo.berkeley.edu.

**Figure 3:**
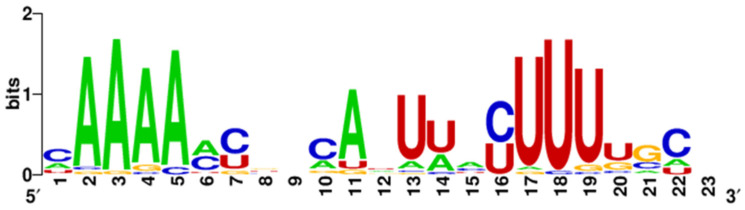
Variability in the miR548-3p strand sequence family. Created by WebLogo on: weblogo.berkeley.edu.

MiR-548—This is a family of microRNA precursor genes specific to primates, with an exceptionally common occurrence in the genome. According to miRbase, 73 miR-548 precursor genes located on almost all human chromosomes have been distinguished, which is why researchers have put forward a hypothesis about the transposon origin of this microRNA. Compared to other microRNAs, this family is characterized by relatively low sequence conservation, which, according to researchers, may lead to the separation of new microRNA families with different purposes and functions in the future [159]. This high heterogeneity of miR-548 strands is presented on Figure 2 and Figure 3 with the size of the letters expressing the frequency of the nucleotides in given location through the whole group of the miR-548 strands. 

In CRC, exosomal miR-548am-5p was characterized by increased expression, and its downregulation resulted in reduced CRC cell proliferation and their ability to form spheroids, as well as increased apoptosis [160]. Its deregulation detected in plasma was also correlated with TNM classification and suggested worse OS [161]. Three members of the miR-548 family—miR-548d-5, miR-548j, and miR548m—are involved in the regulation of PTPN12 [162], whose mutations can induce colon cancer [163]. A relationship between miR-548c-3p and thymidylate synthase (TYMS) and Abcg2 proteins was studied and demonstrated that miR-548c-3p increased susceptibility to 5-FU treatment by limiting the expression of both of them [88]. Abcg2 is one of the key ATP-binding proteins, and its expression is strongly restricted in CRC. No correlation of expression with clinical features of the tumour was found. In addition, it plays an important role in cellular drug transport and their distribution in the body [164]. TYMS is a key enzyme synthesizing TMP in the cell, which is why it is a target for drugs such as 5-FU. Inhibition of TYMS expression has been linked to reduced proliferation and invasiveness of colon cancer cells and has been shown to be due to the regulation of EMT-related proteins via the TYMS/TM4SF4 pathway [165]. Xu et al.’s studies demonstrated the presence of a negative feedback loop between miR-548b and WNT2 (Figure 1). This protein was overregulated in CRC, and its downregulation reduced the malignancy of colon cancer cells [166]. A study conducted on a limited number of postoperative samples showed overregulation of miR-548aa, the deregulation of which significantly influenced the negative clinical features of the tumour [167]. Long non-coding RNA TCONS_00026334 significantly affected the development of colorectal tumours by sponging MiR-548n, which in turn could not bind onco-suppressive TP53INP1, thereby limiting tumour cell proliferation [168]. The results of studies on interferon showed that one of its polymorphisms, T/T rs141112353-, showed a significant association with miR-548ap-3p and tumour cell proliferation and M2 macrophage polarization [169]. Exosomal miR-548c-5p was downregulated in the serum of CRC patients and was shown to be functionally related to glucose metabolism via regulation of phosphoglycerate kinase 1 (PGK1) [170,171]. MiR-548a-3p was also downregulated in SW480 and HCT116 cells, and its activity inhibited tumour growth by targeting oncogenic TPX2, which is involved in microtubule assembly [172]. MiR-548ac inhibits carcinogenesis by acting on TMEM158 and further on the TGFB1/PTEN/AKT and mTOR pathways. This pathway is also affected by miR-548d-3p, acting through EGFR/ERBB2 and PI3K [173].

MiR-607, the gene encoding miR-607-5p, is located on chromosome 10 in the intragenic region. So far, a single original study has been published relating to this microRNA, and it is the result of studies on fecal microRNA markers from 2023. On their basis, it was concluded that miR-607-5p detected in stool could effectively distinguish healthy people from people with colon cancer. Reduced expression of this miRNA indicated the presence of progressive cancer or adenoma. miR607 presented the highest diagnostic accuracy as part of a panel of five marker microRNAs tested together—miR-1246, miR-6777-5p, miR-4488, miR-149-3p, and miR-607-5p. The targets for miR-607-5p are proteins associated with nuclear DNA replication [64].

MiR-1246—The mirBase database contains the sequence of only one mature strand of miRNA-1246, with low certainty of existence due to the insufficient level of knowledge about it and the discussion about the origin of this transcript [174]. The results of previous studies allow us to characterize this miRNA as an oncogenic one, whose targets are the p53, CADM1, CCNG2 or THBS2 pathways. The expression of miR-1246 is increased in CRC and other cancers and is detected in exosomes, so it can be used as a CRC marker as it is significantly associated with OS [64,69,89,175]. Recent studies have also focused on the aspect of the occurrence of RNA isoforms and their different functions and commonness in cells. Comparison of wild-type miR-1246 and its two isoforms, ISO-miR-1246_a and ISO-miR-1246_G, shows that they differ from it by a modified/shifted seed sequence [89]. The consequence of such a change in miRNA, resulting from alternative cleavage of pre-miRNA by Drosha/Dicer or post-transcriptionally by nucleotidyltransferases [176], is different targets and regulation of other pathways than canonical miRNAs. The activity of miR-1246 isoforms affected pathways related to cell proliferation and tumorigenesis, and wild-type miR-1246 targeted proteins of cell cycle pathways. Despite the strong inhibition by miR-1246 of oncogenes such as key regulators of cell cycle and tumorigenesis—*CCND3* and *CCD25A*—upregulation of this miR in colon cancer cells resulted in reduced viability, migration, and apoptosis, due to inhibition of genes *LATS2*, *EREG*, and *PPP2CB* (coding a catalytic subunit for protein phosphatase 2A—PP2A) (Figure 1) which leads to increased cell proliferation and confirming its oncogenic nature [89,90]. It was also detected in exosomes, and then it was confirmed that secretion from cancer cells could affect distant cells. Fibroblasts exposed to miR-1246 transfection were transformed into a CAF-type phenotype—they were activated, and the level of mir1246 expression was increased in them (Figure 1). High levels of miR-1246 were negatively correlated with disease-free time (DFS). This was due to the inhibition of axin and GSK3β in the WNT/β-catenin pathway, thanks to which β-catenin could induce gene expression in fibroblasts. This mechanism also occurred in tumour cells, leading to the formation of metastases, and was amplified by exosomal miR-1246 derived from both other tumour cells and CAFs [177]. MiR-1246 from CRC cells carried in exosomes can also affect hepatic stellate cells by silencing the INSIG1 gene—a regulator of cholesterol and glucose metabolism. Deregulation of these pathways leads to the accumulation of excess cholesterol in cells, resulting in the activation of hepatic stellate cells (HSCs). The effect is the development of changes in the microenvironment in the liver, e.g., the formation of fibrosis. Both the physical change in architecture and the increased level of cholesterol in the microenvironment promote the nesting and growth of metastases of colorectal cancer and other cancers [69]. Studies also report an indirect effect of changes in cholesterol levels on the risk of developing and the course of colon cancer [178].

miR-4772-3p—Its gene is located on chromosome 2 in the non-coding region of the *IL18RAP* gene, an additional protein of the interleukin 18 receptor. In cancer and Alzheimer’s disease, the expression of mir4772 is reduced. The predicted targets are (1) transferrin receptor 1 TFRC, necessary for endocytic iron uptake by the cell, (2) reticulon 4 RTN4I, associated with EMT, cell adhesion, and migration, and (3) RAB9A, which is one of the most important Ras group proteins [91]. Moreover, studies on regulatory T lymphocytes (Terg) in malignant pulmonary effusion have shown that the direct target of miR-4772 is mRNA of *IKZF2* and its protein Helios. It is a transcription factor that is one of the most important tumour suppressors [92]. A strongly reduced (compared to patients without relapse) expression of exosomal miR-4772 in plasma was associated with a worse prognosis for the colon cancer patients and a higher probability of relapse and death. The levels of miR-4772-3p also changed in patients after adjuvant therapy with FOLFOX; however, it was not possible to clearly prove whether miR-4772 can be a reliable marker of treatment response [179]. To this day, this miRNA alongside miR-34a-5p, miR-503-5p, ITGAM, MPO, and MMP9 particles were successfully tested as markers of organ disfunction in sepsis patients, opening the way for prognosis and treatment improvement [180].

MiR-6803-5p—This is located on chromosome 19 in the non-coding region of the *PPP6R1* gene, whose protein is a scaffold for the PP6 subunit and is involved in the TNF-α response pathway. This gene also contains sequences for miR-6802 and miR-6804, on the activity of which there is no literature data yet. The function of miR-6803 in colorectal cancer has been described so far in one original paper [93]. The expression level of this miRNA was found to be increased and negatively correlated with OS, DFS, and the levels of the receptor tyrosine-protein phosphatase O (PTPRO) protein. This enzyme is a transmembrane receptor that is involved in the course of diseases such as Parkinson’s disease or inhibition of metastasis in breast cancer. Mir-6803-5p downregulates its level by activating the NF-κB pathway (Figure 1) and enhancing LPS-induced inflammation by stimulating the synthesis of proinflammatory cytokines. Reduced levels of E-cadherin and increased levels of vimentin have also been reported in HCT116 cells overexpressing miR-6803-5p. This may suggest a link between this microRNA and the induction and regulation of EMT or metastasis [181].

## 5. Conclusions and Future Perspectives

MicroRNAs are among the most important epigenetic regulators, influencing cancer development, immune responses, chronic diseases, and organismal homeostasis. This article discusses selected microRNAs of functional and clinical significance—such as miR-21, miR-29b, miR-148a, and miR-155—with a particular focus on their roles in key signalling pathways, colorectal cancer progression, and their diagnostic and prognostic potential. Due to their high stability and presence in various biological materials (tissue, blood, saliva, stool), miRNAs represent promising candidates for non-invasive diagnostic applications. Although preclinical studies suggest the feasibility of using miRNAs as therapeutic agents, none have yet progressed to phase III clinical trials. Major challenges remain, including molecular instability, the lack of selective delivery to tumour cells, and the risk of off-target effects. Further progress will require the standardization of miRNA isolation and analysis methods—especially in the context of liquid biopsy—and the clinical validation of diagnostic panels in large patient cohorts. Looking ahead, the development of high-throughput technologies and the integration of miRNA data with multi-omics platforms and artificial intelligence may significantly accelerate their clinical implementation.

## Figures and Tables

**Table 1:** Summary table of the main targets, pathways and characteristics of described miRNAs.

miRNA	Type of miRNA	Main Targets	Pathways	Clinical Significance	Detected in:	Source
**miR-21-5p**	onco-miRNA	TGFβR2, CHL1	TGF-β, PI3K/Akt	Diagnostic biomarker, resistance to treatment	Plasma, tissue	[56,57]
**miR-29b-3p**	suppressor	Wnt, TGFβ, MAPK, TIAM1	Wnt/β-catenin, EMT	Prognostic biomarker, increase sensitivity to 5-FU	Plasma, tissue	[58,59]
**miR-148a-3p**	suppressor	SLC7A11, GPX4, MHC I (CANX), WNT10b	Ferroptosis, Wnt	Tumour suppression, immunomodulation	Tissue	[48,60,61]
**miR-149-3p**	suppressor	CYBRD1, HOXB8	Wnt, AKT	Increase sensitivity to 5-FU	Plasma, stool	[62,63,64]
**miR-155-5p**	onco-miRNA	PTEN, FOXO3a, NF-κB	PI3K/Akt, immune response	Regulator of immune systems, metastasis formation, inflammatory response	Exosomes, tissue	[65,66,67]
**miR-194-5p/3p**	suppressor	MAP4K4, KLK10, TGFα, VAPA	MAPK, EMT	Tumour growth and metastasis inhibitor	Tissue	[68,69,70]
**miR-200b-3p**	suppressor	Wnt1, Akt2, ZEB1, E2F3, MAGP2	Wnt/β-catenin, c-Myc	Inhibits metastasis and treatment resistance	Tissue, exosomes	[71,72,73,74]
**miR-204-5p**	suppressor	CREB1, RAB22, B4GALNT2	Wnt/β-catenin, glicosylation	Tumour growth and metastasis inhibitor	Tissue	[75,76,77]
**miR-320a-3p**	suppressor	CDK6, FOXM1, β-catenin, TUSC3	Wnt, MAPK	Diagnostic biomarker (in a panel), chemosensitivity	Plasma, exosomes	[78,79]
**miR-323a-3p**	suppressor	EGFR/Erb3, TYMS, NEK6	PI3K/Akt, EGFR	Increase sensitivity to 5-FU and TKI	Tissue	[80,81,82]
**MiR-376b-3p**	suppressor	SMAD, PRKD1	TGF-β, β-catenin	Prognostic biomarker	Plasma	[83,84]
**miR-382-5p**	suppressor	NRF2, PD-L1, KLF12, HIPK3	Metabolic and metastatic pathways	Inhibits metastases, biomarker	Tissue	[85,86,87]
**miR-548c-3p**	Various	TYMS, ABCG2,	EMT, mTOR, PI3K/Akt	Modulates sensitivity to 5-FU	Tissue, exosomes	[88]
**miR-607-5p**	suppressor	Replications genes	undefined	Diagnostic biomarker (stool miRNA panel)	Stool	[64]
**miR-1246**	onco-miRNA	AXIN, CCND3, INSIG1, p53	Wnt/β-catenin, cell cycle	Promotes metastases	Exosomes, plasma	[67,89,90]
**miR-4772-3p**	suppressor	TFRC, RTN4I, RAB9A, IKZF2	EMT, immunologic regulation	Prognostic and relapse biomarker	Plasma, exosomes	[91,92]
**miR-6803-5p**	onco-miRNA	PTPRO	NF-κB, EMT	Worse prognosis biomarker, correlated with metastases	Tissue	[93]

## Supplementary Materials

The following supporting information can be downloaded at: https://www.mdpi.com/article/10.3390/cancers17132135/s1, Table S1. MiR-21 3p and 5p sequences; Table S2. MiR-29 group 3p and 5p strand sequences; Table S3. miR-148 group 3p and 5p strand sequences; Table S4. MiR-149 3p and 5p strands sequences; Table S5. MiR-155 3p and 5p sequences; Table S6. MiR-194 group 3p and 5p strand sequences; Table S7. MiR-200 group 3p and 5p strand sequences; Table S8. MiR-320 group 3p and 5p sequences; Table S9. MiR-323 group 3p and 5p strand sequences; Table S10. miR-376 group 3p and 5p strand sequences; Table S11. miR-382 3p and 5p sequences; Table S12. MiR-607- 5p strand sequence; Table S13. MiR-1246 5p strand sequence; Table S14. MiR-4772 3p and 5p strand sequences; Table S15. miR-6803 3p and 5p strand sequences.
