# Supplementary material for: Diagnostic and Therapeutic Potential of Selected microRNAs in Colorectal Cancer: A Literature Review
Source: Cancers (Basel). 2025 Jun 25;17(13):2135. doi: 10.3390/cancers17132135 (PMC12248441; doi:10.3390/cancers17132135)
Supplement: Supplementary file 1 [file cancers-17-02135-s001.zip › cancers-3601775-supplementary.pdf]

Table S1. MiR-21 3p and 5p sequences.

Stem-loop precursor	Chromosome	Strand	Sequence of mature miRNA
miR-21	17	3p	CAACACCAGUCGAUGGGCUGU
		5p	UAGCUUAUCAGACUGAUGUUGA

Table. S2. MiR-29 group 3p and 5p strand sequences.

Stem-loop precursor	Chromosome	Strand	Mature miRNA sequence
miR-29a	7	3p	UAGCACCAUCUGAAAUCGGUUA
		5p	ACUGAUUUCUUUUGGUGUUCAG
miR-29b-1	7	3p	UAGCACCAUUUGAAAUCAGUGUU
		5p	GCUGGUUUCAUAUGGUGGUUUAGA
miR-29b-2	1	3p	UAGCACCAUUUGAAAUCAGUGUU
		5p	CUGGUUUCACAUGGUGGCUUAG
miR-29c	1	3p	UAGCACCAUUUGAAAUCAGUGUU
		5p	UGACCGAUUUCUCCUGGUGUUC

Table. S3. miR-148 group 3p and 5p strand sequences.

Stem-loop precursor	Chromosome	Strand	Mature-miRNA Sequence
MiR-148a	7	3p	UCAGUGCACUACAGAACUUUGU
		5p	AAAGUUCUGAGACACUCCGACU
MiR-148b	12	3p	UCAGUGCAUCACAGAACUUUGU
		5p	AAGUUCUGUUAUACACUCAGGC

Table S4. MiR-149 3p and 5p strands sequences.

Stem-loop precursor	Chromosome	Strand	Mature-miRNA Sequence
MiR-149	2	3p	AGGGAGGGACGGGGGCUGUGC
		5p	UCUGGCUCCGUGUCUUCACUCCC

Table S5. MiR-155 3p and 5p sequences.

Stem-loop precursor	Chromosome	Strand	Mature-miRNA Sequence
MiR-155	21	3p	CUCCUACAUAUUAGCAUUAACA
		5p	UUAAUGCUAAUCGUGAUAGGGGUU

Table S6. MiR-194 group 3p and 5p strand sequences.

Stem-loop precursor	Chromosome	Strand	Mature-miRNA Sequence
MiR-194-1	1	5p	UGUAACAGCAACUCCAUGUGGA
MiR-194-2	11	3p	CCAGUGGGGCUGCUGUUAUCUG
		5p	UGUAACAGCAACUCCAUGUGGA

Table S7. MiR-200 group 3p and 5p strand sequences.

Stem-loop precursor	Chromosome	Strand	Mature-miRNA Sequence
MiR-200a	1	3p	UAACACUGUCUGGUAACGAUGU
		5p	CAUCUUACCGGACAGUGCUGGA
MiR-200b	1	3p	UAAUACUGCCUGGUAAUGAUGA
		5p	CAUCUUACUGGGCAGCAUUGGA
MiR-200c	12	3p	UAAUACUGCCGGGUAAUGAUGGA
		5p	CGUCUUACCCAGCAGUGUUUGG
MiR-141	12	3p	UAACACUGUCUGGUAAAGAUGG
		5p	CAUCUUCCAGUACAGUGUUGGA
MiR-429	1		UAAUACUGUCUGGUAAAACCGU

Table S8. MiR-320 group 3p and 5p sequences.

Stem-loop precursor	Chromosome	Sequence of mature miRNA
miR-320a-3p	8	AAAAGCUGGGUUGAGAGGGCGA
miR-320a-5p	8	GCCUUCUCUUCCCGGUUCUUCC
miR-320b-1	1	AAAAGCUGGGUUGAGAGGGCAA
miR-320b-2	1	AAAAGCUGGGUUGAGAGGGCAA
miR-320c-1	18	AAAAGCUGGGUUGAGAGGGU
miR-320c-2	18	AAAAGCUGGGUUGAGAGGGU
miR-320d-1	13	AAAAGCUGGGUUGAGAGGA
miR-320d-2	X	AAAAGCUGGGUUGAGAGGA
miR-320e	19	AAAGCUGGGUUGAGAAGG

Table S9. MiR-323 group 3p and 5p strand sequences.

Stem-loop precursor	Chromosome	Strand	Sequence of mature miRNA
miR-323a	14	3p	CACAUUACACGGUCGACCUCU
		5p	AGGUGGUCCGUGGCGCGUUCGC
miR-323b	14	3p	CCCAAUACACGGUCGACCUCUU
		5p	AGGUUGUCCGUGGUGAGUUCGCA

Table S10. miR-376 group 3p and 5p strand sequences.

Stem-loop precursor	Chromosome	Strand	Sequence of mature miRNA
miR-376a-1	14	3p	AUCAUAGAGGAAAAUCCACGU
		5p	GUAGAUUCUCCUUCUAUGAGUA
miR-373a-2	14	3p	AUCAUAGAGGAAAAUCCACGU
		5p	GGUAGAUUUUCCUUCUAUGGU
miR-376b	14	3p	AUCAUAGAGGAAAAUCCAUGUU
		5p	CGUGGAUAUUCCUUCUAUGUUU
miR-376c	14	3p	AACAUAGAGGAAAUUCCACGU
		5p	GGUGGAUAUUCCUUCUAUGUU

Table. S11. miR-382 3p and 5p sequences.

Stem-loop precursor	Chromosome	Strand	Sequence of mature miRNA
miR-382	14	3p	AAUCAUUCACGGACAACACUU
		5p	GAAGUUGUUCGUGGUGGAUUCG

Table S12. MiR-607- 5p strand sequence.

Stem-loop precursor	Chromosome	Strand	Sequence of mature miRNA
Mir-607	10	5p	GUUCAAAUCCAGAUCUAUAAC

Table. S13. MiR-1246 5p strand sequence.

Stem-loop precursor	Chromosome	Strand	Sequence of mature miRNA
MiR-1246	2	5p	AAUGGAUUUUUGGAGCAGG

Table S14. MiR-4772 3p and 5p strand sequences.

Stem-loop precursor	Chromosome	Strand	Sequence of mature miRNA
miR4772	2	3p	UGAUCAGGCAAAAUUGCAGACU
		5p	CCUGCAACUUUGCCUGAUCAGA

Table. S15. miR-6803 3p and 5p strand sequences.

Stem-loop precursor	Chromosome	Strand	Sequence of mature miRNA
miR-6803	19	3p	UCCCUCGCCUUCUCACCCUCAG
		5p	CUGGGGGUGGGGGCUGGGCGU
